# Book Review

**Published:** 1907-06

**Authors:** 


**Applied Bacteriology.** By CRESACRE G. MOOR, M.A., F.I.C., with the co-operation of R. T. HEWLETT, M.D., D.P.H. Third Edition. Pp. 475. London: Baillière, Tindall & Cox. 1906.—The sub-title of this work—*An Elementary Handbook for the Use of Students of Hygiene, Medical Officers of Health, and Analysts*—indicates its scope. Its usefulness is demonstrated by the appearance of the Third Edition. The subject-matter has been brought well up to date, and the ground covered is sufficient for ordinary class work. In addition to micro-organisms, ferments are considered. There are chapters also on "Disinfection and Disinfectants" and on "Water, Air, &c." The illustrations are a little crude, and the thick paper makes the book inconveniently bulky.

**Lectures on Midwifery for Midwives.** By A. B. CALDER, M.B. Pp. xi., 274. London: Baillière, Tindall & Cox. 1906.—These lectures given in rather discursive style read very well, and most of the points required for a midwife are clearly indicated. In some of the chapters, especially those on mechanisms, it might have been easier to grasp the subject if it had been tabulated. It is always difficult to follow a description, even when in good English, which runs easily from one stage to another without definitely marking off the other. The chapter on the child and its feeding is good and rational. A very useful lecture would have been one on those cases in which it is advisable to send for a doctor, this being one of the most important points in which to instruct the midwife. The application of the Midwives' Act is clearly set out, and should be useful. Auscultation is to be practised only by the ear, in many cases a wooden stethoscope is much more valuable. The plates, although small and crowded, are on the whole very useful. We think the book will certainly find a well-merited sale.

**Transactions of the American Pediatric Society.** Vol. XVII. New York: E. B. Treat and Co. 1906.—There is good scientific work exhibited in this volume. Congenital laryngeal stridor is shown to be due to a folded gutter-like epiglottis, which leaves merely a chink for breathing; this condition is always present to some extent in young children, and when excessive stridor appears it must not be confused with "thymic asthma." Very early gastro-enterostomy is favoured for congenital pyloric stenosis. Citrate of soda, three grains to the ounce of milk, is very useful in intestinal dyspepsia. Intubation for diphtheria should be performed earlier than is usual. Cortical decapsulation of the kidneys for severe chronic nephritis shows better results in children than adults. Milky pleural effusions, when not due to chyle or degenerated fat, are caused by altered albumins and globulins. Several of the other papers are worth reading.

**A Preliminary Inquiry concerning the Milk Supply of Schools.** By C. E. Shelly, M.D. Pp. 11. London: J. & A. Churchill. 1906.—This is a report on an inquiry, inaugurated by the Medical Officers of Schools Association, with a view to determining what statistical evidence existed either for or against the practice of boiling or sterilising the milk supplied to schools. In response to a circular forty-three replies were received, of which forty-one have been tabulated. The figures obtained are curious, but although the returns include observations on a large number of children, the figures are not really sufficient for the purpose for which they were collected. If any lesson is to be learnt, it is that we should look rather towards the securing of a pure milk supply for protection against epidemic disease than attempt to guard against infection by boiling or otherwise treating the milk. The figures are handled dexterously but with much caution by the author, and the pamphlet will be found to be interesting and suggestive to those concerned in the feeding of children at boarding schools.

**A Manual of Diseases of the Eye.** By Charles H. May, M.D., and Claud Worth. Pp. viii., 400. London: Baillière, Tindall & Cox. 1906.—This is an excellent text-book for the medical student; it covers the ground well, and is short and concise. The statements contained are given in a crisp manner, and so are likely to remain in the mind of the reader, but on this account the perusal of the book is rather like reading a dictionary; still, also like a dictionary, it is full of information, and can be confidently recommended to the student or practitioner who is in a hurry for his knowledge.

**A Handbook of Diseases of the Nose and Pharynx.** By James B. Ball, M.D. Fifth Edition. Pp. xii., 388. London: Baillière, Tindall & Cox. 1906.—A text-book which has rapidly run through four editions obviously has proved acceptable to students and practitioners, and it becomes all the more essential that the work should be accurate and up to date. The author has evidently spared no pains to make the many alterations and corrections that so soon become necessary in a relatively young and developing speciality, and yet without materially adding to the size of the book, which is worthy of the author's reputation.

**Davos as Health Resort.** Davos: Davos Printing Company, Ltd. 1906.—This book of 316 pages, forwarded to us by the Davos Public Interests Association, consists of a series of articles written by the best authorities in Davos, and with an introduction written by Dr. W. R. Huggard, H.B.M. Consul. The contributors, sprung from many different lands in Europe, have made Davos their more or less permanent home, and have acted in friendly co-operation in producing a book which gives the best information, historical, topographical, geological, ethnological, botanical, climatic, physiological, pathological and sociological, of a place which is of profound interest to those who have visited it, and will be of interest to those who read about it. Dr. Huggard commends the volume to his brethren at home, and trusts that they will find it as interesting as they will assuredly find the place when they make its acquaintance for themselves.

**The Climate of Lisbon, Mont Estoril and Cintra.** By Dr. D. G. Dalgado. Pp. viii., 50. London: H. K. Lewis. 1906. —This paper is an echo of the Fifteenth International Congress, held at Lisbon in April, 1906. It is in praise of two health resorts, and its object is to show: (1) That the climate of Lisbon in winter is not variable; (2) that Mont Estoril, as a winter health resort, is in many respects superior to Biarritz, Nice and Catania; and (3) that Cintra, "the most blessed spot in the habitable globe," of Southey, is a very desirable and charming health resort in summer, but not in winter. Those of us who visited Mont Estoril last spring can well realise that the Riviera of Portugal deserves fully to make a reputation for itself as a climatic health resort. Its salubrity has been known for ages, for the monks, excellent judges of a genial climate and of good cheer, selected Estoril and Cascaes for their monasteries. It is now a very popular sea-bathing summer resort, but endeavours are being made to extend its popularity through the winter also.

**The Philippine Journal of Science.** Edited by Paul C. Freer, M.D., Ph.D. Manila. 1906.—We have received Part VI. of this new journal, published by the Bureau of Science of the Government of the Philippine Islands. The inhabitants of these islands may be congratulated on the fact that they are able to produce such an excellent periodical, which must at once take a very high position amongst the scientific journals of the world. This may be accepted as the best evidence of good government, and one of the results arising from the good influence of the United States in their eastern colonies. The article on rinderpest, from the serum laboratory of the Bureau of Science, shows that the Government is using every effort to eradicate the disease. Those which follow, on geological and botanical topics, are illustrated by means of an excellent series of photographic plates. It will be difficult to maintain the standard of this and many other of the publications of the Bureau of Government Laboratories of Manila.

**On Physical Training in Schools,** by W. P. Herringham, M.D., and **The Influence on National Life of Military Training in Schools,** by T. C. Horsfall. Pp. 12. London: J. and A. Churchill. 1906.—These essays are issued by the medical officers of Schools Association, and give very terse and useful evidence in support of the views of the National League for physical education and improvement. Dr. Herringham remarks that all boys and girls ought to go through systematic physical drill, and ought to be taught that it is their proper ambition to develop their bodies and to be as well grown and strong as they can. He quotes Almond, who used to speak of it as a religious duty, and of loafing and bad hygiene as physical sin. Mr. Horsfall is convinced "that very great good would come to English children and to the whole community from the introduction of military drill and instruction in shooting into all our schools, and that there would be no drawbacks to the advantages which it would give in improvement of health, increase of public spirit and patriotism, and of increase also of the safety of the nation."

**Sound and Rhythm.** By W. Edmunds. Pp. 96. London: Baillière, Tindall and Cox.—Children in primary schools being taught singing, the author has written this little book to suggest that it would be to their advantage to be also taught the mechanism of sound and hearing. The book is written as clearly and simply as possible; still we cannot help thinking that much of it, and specially the chapter on musical scales, would be beyond the comprehension of, at any rate, young children. The first two chapters deal with the production and conduction of sound and with the wave form of sound vibration. The third chapter treats of musical scales and intervals, harmonies, consonance, dissonance, and beat tones, &c.; the fourth with organ pipes and resonance in tubes, and the fifth with "time" and movement as illustrated by different metre in poetry and in dancing. Chapter VI. deals with the anatomy and physiology of the ear, and connected with it are a series of models, those of the ossicles much enlarged being quite good; those of the temporal bone showing inner wall of tympanum, and ossicles with drum being quite useless, at any rate for those unacquainted with its anatomy. The book ends with a short chapter on the production of the voice and finally a description of the models. The subject of the book is an extremely interesting one, and we doubt if it would be possible to deal with it in a more lucid fashion. A number of illustrations add considerably to the value of the book.

**Supplementary Essays on the Cause and Prevention of Dental Caries.** By J. SIM WALLACE, M.D., D.Sc., L.D.S. Pp. vii., 81. London: Baillière, Tindall and Cox. 1906.—This is a book that every practitioner, medical and dental, should read with interest. It is very possible that he may not agree with much that the writer puts so forcibly and quaintly, but the book will make him think. It is calculated to disturb the complacency of anyone who imagines he knows all about dental caries. There is a most healthy want of reverence for generally received opinions.

**A System of Surgical Nursing.** By A. N. M'GREGOR, M.D. Pp. xi., 554. Glasgow: David Bryce and Son. [1905.]—This is a large work of 550 pages. There are a very large number of works on this subject now to be obtained, and it is hard to advise a nurse as to which is the best for them to get. Many are too much devoted to surgery, but this is quite an exception in this particular. Although the main surgical points are mentioned, there is in each case a fully detailed account of the nursing aspect. The chapter on dressings and on the preparation for operation are very good and concise. A few words on the removal of plaster of Paris would have been of advantage, as it is sometimes a very difficult process. It often happens that a breast has to be emptied of its milk, a proceeding requiring great care and experience, and it is therefore a pity that the detail of this proceeding has been omitted. There are no illustrations, though in a work of this sort we doubt whether this is a disadvantage. We are rather surprised that no mention has been made of the detail required in private nursing, in fact the subject is not mentioned. Undoubtedly the book is a very valuable one, and we should think is assured of a large sale.

**Practical Nursing.** By ISLA STEWART and HERBERT E. CUFF, M.D. New Edition. Pp. viii., 436. London: William Blackwood and Sons. 1904.—We have seldom met a book which has upheld its title as well as this volume. It is practical from beginning to end—no mere list of duties strung together, but an entirely readable accumulation of facts written in good English and in excellent style. We have no desire to criticise the subject matter; it is what it is meant to be, and undoubtedly every nurse should read it. There are a few illustrations, and rather curious ones have been chosen—Buller's shield and Leiter's coils, both very seldom used. Perhaps it is for this reason that they have been selected; also two figures showing arteries being compressed in a somewhat awkward manner. We would advise medical students to look through this work; it is intensely interesting reading, and is sure to be appreciated.

**Le Livre de la Sage-Femme et de la Garde.** By Dr. R. De Seigneux. Pp. 62. Genève: Henry Kündig. 1905.—This is a short hand-book for midwives published in Geneva, the author being a *privat-docent* of the University. It is an excellent short practical manual, and is the more valuable since the author has incorporated with it a series of hints on the early symptoms and treatment of cancer of the uterus. While the instructions given in it are excellent and practical, we think that it errs on the side of brevity in one or two important particulars. For example, we do not think that the treatment of post-partum hemorrhage is sufficiently dealt with by instructing the midwife to massage the uterus, give ergot, and send for a medical practitioner. This complication is so dangerous and often so sudden that the directions for its treatment cannot be too full or too exhaustive. All possible measures should be described, and the order in which they are to be applied should be laid down. In a case of this kind the patient's life depends on the prompt application of the proper remedies by the person on the spot, and the midwife should not be confined to the measures described, since, if they should prove ineffectual, medical aid would arrive too late to save the patient. The hints as to the early symptoms of cancer of the uterus are most valuable, and cannot be too widely disseminated. A series of capital forms for case-taking are embodied in the book.

**Lectures upon the Nursing of Infectious Diseases.** By F. J. Woollacott, M.A., M.D. Pp. vii., 147. London: The Scientific Press, Limited. 1906.—Most books on nursing are too apt to be a dissertation on the various diseases with but little on the actual nursing. In this work the reverse is the case. For this reason it is a book of considerable value. True, the symptoms are stated and a very good account of the disease is given, but it is concise, and it is followed by nursing instructions which on every page show that the author has evidently not only an intimate knowledge of the practical side of the cases, but also he has been able to collect just those little points in nursing which it is generally so hard to get out of a book. We would recommend it highly to nurses and even to medical men.

